# Effects of diet and antihyperglycemic drugs on erectile dysfunction: A systematic review

**DOI:** 10.1111/andr.13192

**Published:** 2022-05-13

**Authors:** Giuseppe Defeudis, Rossella Mazzilli, Alfonso Maria Di Tommaso, Virginia Zamponi, Francesco Carlomagno, Dario Tuccinardi, Mikiko Watanabe, Antongiulio Faggiano, Daniele Gianfrilli

**Affiliations:** ^1^ Unit of Endocrinology and Diabetes, Department of Medicine University Campus Bio‐Medico di Roma Rome Italy; ^2^ Unit of Endocrinology, Department of Clinical and Molecular Medicine Sapienza University of Rome Rome Italy; ^3^ Department of Experimental Medicine, Section of Medical Pathophysiology, Food Science and Endocrinology Sapienza University of Rome Rome Italy

**Keywords:** antihyperglycemic drugs, diabetes mellitus, diet, erectile dysfunction, sexual function

## Abstract

**Background:**

Erectile dysfunction is recognized as one of the complications of diabetes mellitus. To date, a wide gap of knowledge is present on the efficacy of pharmacological treatments of diabetes mellitus on erectile function, acting not only through metabolic control. Similarly, the effects of different diet regimens on erectile dysfunction are still debated.

**Objectives:**

We aimed to explore the effects of diet and antihyperglycemic drugs, considering both old and novel therapeutic approaches, on erectile function.

**Materials/methods:**

We performed a systematic review, following the PRISMA guidelines. The research was conducted on studies reporting erectile dysfunction assessment in subjects with diabetes and the relationship with diet and antihyperglycemic drugs.

**Results:**

The Mediterranean diet was effective in most studies for the protection of erectile function. Furthermore, antihyperglycemic drugs seem to show an overall protective role on erectile function.

**Discussion/conclusion:**

Although encouraging results are present for all classes of antihyperglycemic drugs, several studies are needed in humans, mainly on acarbose, pioglitazone, dipeptidyl‐peptidase‐4 inhibitors, and sodium‐glucose cotransporter‐2 inhibitors.

## 1 INTRODUCTION

Erectile dysfunction (ED) is the inability to achieve or maintain penile erection sufficient to obtain satisfactory sexual activity.^1^, ^2^ The prevalence of ED increases with age.^1^, ^2^ ED can be due to many factors: psychological, hormonal, atherosclerotic (due to endothelium‐dependent vasodilatation impairment, increased basal and myogenic tone of corpus cavernosum smooth cells, and reduced penile arterial lumen^3^, ^4^), drug‐related (e.g., antihypertensive or psychotropic medications),^5^ and systemic diseases.

Among endocrine dysfunctions, diabetes mellitus (DM), both type 1 and type 2 (T1DM and T2DM),^6^, ^7^ hypogonadism,^8^, ^9^, ^10^, ^11^, ^12^ obesity,^13^ and insulin resistance,^14^ play an important role in the pathogenesis of ED.^14^, ^15^, ^16^ DM is one of the most common chronic diseases due to impaired carbohydrate metabolism. Alongside insulin therapy, which is the gold standard in T1DM patients, or educational therapy for all subjects with DM,^17^ new antihyperglycemic drugs have been introduced in recent decades for the treatment of DM.^18^ Therefore, along with diet, metformin, sulfonylureas, glinides, and thiazolinediones (TZD), the following drugs are now widely used for the management of DM: dipeptidyl‐peptidase‐4 (DPP‐4) inhibitors, glucagon‐like peptide‐1 (GLP‐1) receptor agonists (RAs), and sodium‐glucose cotransporter‐2 (SGLT‐2) inhibitors.^18^ To date, some studies evaluated the effects of DM management on sexual dysfunction in males and females,^19^, ^20^ but the data are still limited on ED.

The aim of this work was to systematically review the effect of diet and antihyperglycemic drugs, both considering old and new therapeutic approaches, on ED.

## 2 MATERIALS AND METHODS

**FIGURE 1:**
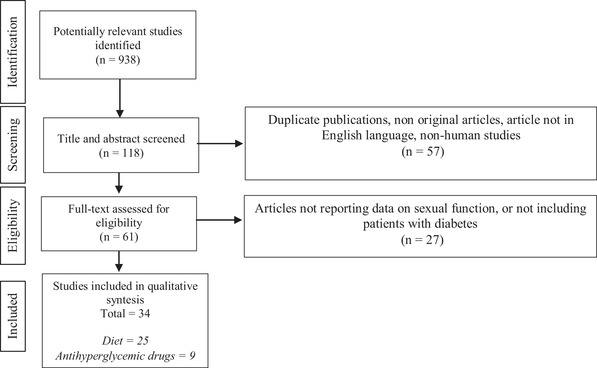
PRISMA guidelines

We followed the PRISMA guidelines in this systematic review (Figure 1).

### 2.1 Article identification

We searched the PubMed, Science Direct, and Google Scholar databases for studies reporting ED assessment in diabetic patients and the relationship with diet and antihyperglycemic drugs up to February 20, 2022.

The search terms used were: “erectile dysfunction and antihyperglycemic drugs;” “erectile dysfunction and metformin;” “erectile dysfunction and diet;” “erectile dysfunction and insulin;” “erectile dysfunction and sulfonylureas;” “erectile dysfunction and glinides;” “erectile dysfunction and thiazolidinediones;” “erectile dysfunction and pioglitazone;” “erectile dysfunction and GLP‐1;” “erectile dysfunction and glucagon‐like peptide‐1;” “erectile dysfunction and liraglutide;” “erectile dysfunction and lixisenatide;” “erectile dysfunction and exenatide;” “erectile dysfunction and dulaglutide;” “erectile dysfunction and semaglutide;” “erectile dysfunction and acarbose;” “erectile dysfunction and DPP4;” “erectile dysfunction and inhibitors of dipeptidyl‐peptidase‐4;” “erectile dysfunction and sitagliptin;” “erectile dysfunction and vildagliptin;” “erectile dysfunction and saxagliptin;” “erectile dysfunction and linagliptin;” “erectile dysfunction and gemigliptin;” “erectile dysfunction and anagliptin;” “erectile dysfunction and teneligliptin;” “erectile dysfunction and alogliptin;” “erectile dysfunction and trelagliptin;” “erectile dysfunction and omarigliptin;” “erectile dysfunction and sodium‐glucose cotransporter‐2 inhibitors;” “erectile dysfunction and SGLT2;” “erectile dysfunction and empaglifozin;” “erectile dysfunction and dapaglifozin;” “erectile dysfunction and ertuglifozin;” and “erectile dysfunction and canaglifozin.”

We included only English‐language studies on humans, without time restriction and with the following designs: randomized clinical trials, non‐randomized trials, retrospective studies, and case reports. Review articles and meta‐analyses were also screened.

The inclusion criteria were: (1) articles reporting data on the effect of diet on ED; (2) articles reporting data on the effect of antihyperglycemic drugs on ED; (3) studies on humans. The exclusion criteria were: (1) non‐English‐language articles; (2) non‐human studies.

Studies on animal were only considered to describe the possible effect of drugs on erectile function (EF).

### 2.2 Articles selection

Four authors screened articles by abstract and title and the eligible studies were evaluated by retrieving full‐text articles (Rossella Mazzilli, Alfonso Maria Di Tommaso, Mikiko Watanabe, and Virginia Zamponi). Then, two authors extracted data from the articles that fulfilled the inclusion criteria (Giuseppe Defeudis and Dario Tuccinardi). A standardized form was used to extract the following information: first author, year of publication, study design, number of patients enrolled, sexual function assessment, and effect on sexual function.

From the original number of 938 articles, 61 were selected on the basis of title and abstract. Finally, after full‐text examination, a total of 34 articles were included (Tables 1 and 2).

**TABLE 1:** Effect of diet on erectile function (EF) in human studies

Authors	Diet	Diet assessment	Study design	ED assessment	Cases	Type	Effect
Cassidy et al. 2016	Habitual diet	Food‐frequency questionnaires	Prospective cohort study	Five‐point scale	25,096	Healthy men	Higher habitual intake of specific flavonoid‐rich foods is associated with reduced ED incidence
Bauer et al. 2020	Habitual diet	Food‐frequency questionnaires	Prospective cohort study	Five‐point scale	21,469	Healthy men	Adherence to healthy dietary patterns was associated with a lower risk for ED
liu et al. 2016	Habitual diet	Food‐frequency questionnaires	Prospective cohort study	IIEF‐5	1564	Elderly men	Fruit and vegetable consumption had no significant association with the score change of ED
Wang et al. 2013	Habitual diet	Food‐frequency questionnaires	Cross‐sectional study	Single question y/n	1466	Men with T2DM	10% risk reduction of ED was found with each additional daily serving of fruit/vegetable consumed
Chen et al. 2016	Habitual diet	Food‐frequency questionnaires	Cross‐sectional study	IIEF‐5	4208	Men >50 years old	Vegetable intake (*p* = 0.001), and milk and dairy product intake (*p* = 0.001) decreased the IIEF‐5
Fantus et al. 2020	Habitual diet	2‐day food diary	Cross‐sectional study	Five‐point scale	4027	Men 18–85 years	No association between specific diets (low fat, med, unrestricted) and EF
Lu et al. 2021	Habitual diet	Food‐frequency questionnaires	Cross‐sectional study	IIEF‐5	116	Young healthy men <45 years	Plant‐based diet was not associated with ED
Carto et al. 2022	Habitual diet	Food‐frequency questionnaires	Cross‐sectional study	Five‐point scale	2549	Men 20–70 years	Healthful plant‐based diet is associated with less chance of having ED
Huynh et al. 2020	Habitual diet	Dichotomic questionnaire	Cross‐sectional study	IIEF‐5	271	Men accessing a men's health clinic	Men with ED less likely to report an organic diet or intermittent fasting
Giugliano et al. 2010	Habitual diet	Food‐frequency questionnaires	Cross‐sectional study	IIEF‐5	555	Men 35–70 years with T2DM	Greater adherence to Mediterranean diet is associated with a lower prevalence of ED
Angelis et al. 2020	Habitual diet	Food‐frequency questionnaires	Cross‐sectional study	SHIM‐5	150	Men with stable heart failure	The SHIM‐5 score was positively correlated with the Med‐Diet score (*p* = 0.006)
Della camera et al. 2017	Habitual diet	Questionnaire adherence to med diet	Case–control	IIEF‐15	141	Healthy men aged 50–72 years	Patients with ED have lower Med‐Diet scores
Esposito et al. 2006	Habitual diet	Food‐frequency questionnaires	Case–control	IIEF‐5	200	Healthy men with and without ED	Intake of fruits and nuts, and the ratio of monounsaturated lipids to saturated lipids
Mykoniatis et al. 2018	Habitual diet	Food‐frequency questionnaires	Case–control	IIEF‐15	350	Healthy young men	Increased intake of fruits, vegetables, and flavonoids decreases the risk of ED in young men
Lu et al. 2021	Habitual diet	Food‐frequency questionnaires	Case–control	IIEF‐5	184	Men with and without ED (older) including T2DM	Plant‐based diet was associated with a reduced presence of ED and less severe ED
Talib et al. 2015	Intermittent fasting (Ramadam)	Not monitored	Observational study	IIEF (1–5–11–12–15)	45	Men observing Ramadam	No statistically significant differences were found on EF
Ramirez et al. 2016	Habitual diet	14‐item food questionnaire	Observational study	IIEF‐15	440	Men over 40 non‐diabetic with or without ED	Consumption of nuts (>twice a week) (OR 0.41, 95% CI 0.25–0.67) and vegetables (≥once a day) (OR 0.47, 95% CI 0.28–0.77), were inversely related to ED
Khoo et al. 2011	VLCD versus high‐protein low‐fat low‐carbohydrate diet higher in calories	Daily food diaries	RCT	IIEF‐5	31	Men with obesity and T2DM	Similar results for both diet despite different degree of weight loss
Khoo et al. 2010	VLCD versus control	Daily food diaries	Controlled clinical trial	IIEF‐5	68	Obese men with or without T2DM	The degree of weight loss was significantly associated with improvements in EF (*r *= −0.26)
Moran et al. 2016	Higher protein low‐fat diet versus higher carbohydrate low‐fat diet	3‐day food diaries	RCT	IIEF‐15	118	Men with obesity or overweight	No improvement in either group despite significant weight loss
Esposito et al. 2004	Hypocaloric diet versus control	3‐day food diaries	RCT	IIEF‐5	110	Men with obesity and ED	Weight loss and physical activity associated with improvement
Wing et al. 2010	Hypocaloric diet versus control	Daily food diaries	RCT	IIEF‐15	306	Overweight obese men with T2DM	Weight loss intervention was mildly helpful in maintaining EF
Collins et al. 2013	Hypocaloric diet versus control	Follow‐ups and weight loss monitoring	RCT	IIEF‐5	145	Overweight or obese men	Weight loss associated with improvement
Esposito et al. 2006	Mediterranean diet versus control	3‐day food diaries	Controlled clinical trial	IIEF‐5	65	Men with MS and ED	Consumption of a Mediterranean‐style diet in men with the MS and ED at baseline produced significant improvement of EF

Abbreviations: CI, confidence interval; ED, erectile dysfunction; IIEF, International Index Erectile Function; MS, metabolic syndrome; OR, odds ratio; RCT, randomized control trial; SHIM, sexual health inventory for men; T2DM, type 2 diabetes mellitus; VLCD, very‐low‐calorie diet.

**TABLE 2:** Effect of antihyperglycemic drugs on erectile function (EF) in human studies

Class	Drugs	Authors	Study design	ED assessment	Cases	Effect
Biguanide	Metformin	Rey‐Valzacchi et al. 2011	Prospective, randomized, controlled, double‐blind placebo study	IIEF‐5	30	IIEF score increased after metformin treatment
Biguanide and sulfonylurea	Metformin versus glibenclamide	Al‐Kuraishy et al. 2016	Cross‐sectional study	SHIM	91	Sulfonylureas have a better effect on EF
Insulin	CSII versus MDI	Maiorino et al. 2016	Observational study	IIEF‐5	224	Insulin improves EF without statistically significant difference between CSII and MDI
		Kesavadev et al.	Prospective study	IIEF‐5	46	At 6 months, CSII showed increased IIEF‐5 (*p *= 0.0037). More subjects in the CSII than the MDI arm achieved reductions in ED severity
	Intensive insulin treatment versus conventional therapy	Wessels et al. 2011	Cohort study	IIEF	571	Intensive insulin treatment is associated with a decrease incidence of ED in patients with microalbuminuria or non‐proliferative retinopathy, whereas no difference was observed between conventional therapy toward intensive in patient in primary prevention
Thiazolidinediones	Pioglitazone	Gholamine et al. 2008	Prospective, randomized, placebo‐controlled, double‐blinded trial study	IIEF‐6	38	Improved EF and increased sildenafil response
DPP‐4 inhibitors	Linagliptin	Mourad et al. 2019	Prospective study	–	9	Increase tadalafil plasma concentration
GLP‐1 RAs	Liraglutide versus metformin	Giagulli et al. 2015	Retrospective observational study	IIEF‐15	43	Improve of EF, glycemic control and weight loss
	Dulaglutide	Bajaj et al. 2021	Randomized, double‐blind, placebo‐controlled study	IIEF‐15	3725	Improve of EF

Abbreviations: CSII, continuous subcutaneous insulin infusion; DPP‐4, dipeptidyl‐peptidase‐4; ED, erectile dysfunction; GLP‐1, glucagon‐like peptide‐1; IIEF‐5, International Index Erectile Function; MDI, multidrug injection; SHIM, sexual health inventory for men.

## 3 RESULTS

### 3.1 Diet

Dietary habits may have a beneficial or detrimental impact on EF through several mechanisms. The most investigated and solid benefits derived from a healthy diet are antioxidant and anti‐inflammatory mechanisms.

Diets rich in plant foods, and the Mediterranean diet in particular, are characterized by significant intake of polyphenols, which are capable of increasing nitric oxide (NO) availability.^21^ An increase in endothelial progenitor cells could also be involved.^22^ NO availability may also be increased by fish‐derived omega‐3 fatty acids, possibly another mechanism mediating improved EF by the Mediterranean diet.^23^ Moreover, plant‐based diets are rich in antioxidants, exerting protective roles against reactive oxygen species (ROS).^24^ The microbiome may also play a role: a healthy microbiome in fact leads to reduced inflammation, gut permeability, and overall to metabolic improvement, with expected amelioration of ED symptoms.^25^ Conversely, animal and epidemiological studies both highlight a strong detrimental effect of western diets in determining ED, especially when T2DM is coexistent. Hypercaloric diets lead to endothelial damage, establishing ED in rodent models^26^; high‐fat diets increase ROS production in the corpus cavernosum of diabetic rats^27^; high salt intake impairs EF beyond its impact on blood pressure in rats.^28^, ^29^ Animal products are capable of increasing ROS formation^24^ and trimethylamine N‐oxide levels through microbiome modulation with a consolidated role in atherosclerosis pathogenesis, at the root of ED.^25^ Exercise and caloric restriction were reported to improve EF in diabetic^30^, ^31^ or to maintain function in aging rodents.^32^, ^33^

#### 3.1.1 Habitual diet

Several prospective cohort, cross‐sectional, and case–control human studies have been conducted to assess the association between dietary habits and EF, mainly assessed through the International Index of Erectile Function‐5 (IIEF‐5) questionnaire or a five‐point Likert scale (Table 1). Overall, a higher intake of fruits and/or vegetables was associated with lower odds of having or developing ED in most,^34^, ^35^, ^36^, ^37^, ^38^ but not all studies.^39^, ^40^ Interestingly, a study conducted by Chen et al.^41^ reported that a higher intake of vegetables was also associated to worse ED. These findings could be explained by the high use of pesticides in China and Hong Kong, where all three studies reporting neutral or negative results were conducted, although this hypothesis cannot be confirmed. More general concepts of healthy dietary habits^42^ or organic food consumption^43^ were also reported to be associated with a lower prevalence or development of ED, as was adherence to the Mediterranean diet, most commonly assessed through food‐frequency questionnaires.^44^, ^45^, ^46^, ^47^ Flavonoid‐rich foods^35^, ^48^ and nuts^38^, ^47^ were also consumed more frequently by those who did not have ED, likely reflecting a Mediterranean dietary pattern. When a 2‐day food diary was utilized to frame the dietary habits of enrolled patients, adherence to a Mediterranean, low‐fat or unrestricted diet was not associated with different EF^49^; however, a 2‐day food diary is likely a poor tool to evaluate the adherence to dietary patterns, possibly hindering the validity of the results.

#### 3.1.2 Mediterranean diet

Two studies conducted by the same group investigated the effect of an unrestricted Mediterranean pattern compared with control or low‐fat diets in men with either metabolic syndrome and ED or with newly diagnosed T2DM. Men with pre‐existing ED reported an improvement as reflected by an increase in the IIEF‐5 score over 2 years,^50^ whereas those with newly diagnosed T2DM (and no baseline ED) following a Mediterranean dietary pattern reported a smaller decline in sexual function over the course of 8 years.^51^

#### 3.1.3 Low‐calorie diet

Weight loss obtained through a hypocaloric regimen led to significant improvements in the IIEF scores compared with control in men with obesity and T2DM and/or ED.^52^, ^53^, ^54^ When several dietary regimens were compared, leading to similar weight loss, a significant and comparable improvement in the IIEF score was observed in both groups, likely dependent on weight loss. Of note, one study conducted by Moran et al.^55^ did not report any improvement when comparing a higher protein, low‐fat and a higher carbohydrate, low‐fat diet for 1 year in overweight or obese individuals. This could be due to the specific dietary regimens studied, both different from the Mediterranean pattern, or simply because the patients were otherwise healthy and did not show ED at baseline.

#### 3.1.4 Intermittent fasting

Two studies observed the potential impact of intermittent fasting, showing no^56^ or some potential association with decreased odds of ED.^43^ Noteworthy, the first study was conducted on men observing Ramadan, and it is likely that the dietary habits were not necessarily healthy when non‐fasting. Moreover, for obvious reasons, the study was short, possibly hampering the eventual beneficial effects. Conversely, the second study was a survey conducted on patients accessing a men's health clinic, and the reported intermittent fasting was more likely to be framed in a healthier lifestyle, although this was not further investigated in the study.

#### 3.1.5 Very‐low‐calorie diet

Finally, Khoo et al.^57^, ^58^ conducted two studies aiming at evaluating the effect of a very‐low‐calorie diet (VLCD) on EF in men with obesity and T2DM and found that weight loss was significantly associated with improvements in EF.

Overall, a healthy lifestyle similar to the Mediterranean diet is capable of maintaining EF and improving ED to some extent. If weight loss is beneficial in improving cardiovascular health, including EF, it is unclear whether specific dietary regimens prove better than others, independent of weight loss.

### 3.2 Metformin

Metformin is an oral biguanide and the first‐line therapy in patients with T2DM; the antihyperglycemic effect is obtained by increasing peripheral insulin sensitivity and through the inhibition of hepatic gluconeogenesis.^59^ Patel et al.^3^ observed that metformin can improve endothelial‐dependent vasodilatation and decrease sympathetic activity. A recent study conducted by Tseng et al.,^60^ showed that metformin can also improve blood pressure and, consequently, atherosclerosis.

Several studies conducted on animals showed a positive effect of metformin on EF. In this regard, Labazi et al.^61^ studied the effects of metformin on angiotensin II (AngII)‐induced ED in rats. The authors observed that AngII can induce ED by increasing contraction and decreasing relaxation of corpus cavernosum smooth muscle cells; however, metformin can restore this effect.^61^ Metformin could determine this improvement in ED by decreasing extracellular signal‐regulated kinase 1/2 phosphorylation and increasing endothelial nitric oxide synthase (eNOS) phosphorylation in the corpus cavernosum.^61^ Furthermore, Katakam et al.^62^ studied insulin‐resistant rats treated with metformin and observed an enhanced vascular function through improvement of acetylcholine‐induced relaxation. Silva et al.^63^ observed that rats with obesity and insulin resistance have impaired vasomotor function which is improved after metformin administration. In particular, metformin restores the increase in intracavernosal pressure (ICP), improves the relaxation of the corpus cavernosum in response to acethylcoline, and restores the contractile responses to phenylephrine.^63^ The importance of insulin resistance in endothelium function impairment has also been studied by Kim et al.^64^ In particular, the authors observed adipose visceral tissue in the penis of obese rats. Consequently, endothelial (e) and neuronal (n)NOS are decreased. On the other hand, after metformin administration, the adipose tissue decreases/disappears, while nNOS and eNOS levels are restored.^64^ The authors also observed an increase in adenosine monophosphate (AMP)‐activated protein kinase after metformin treatment,^64^ which enhances both nNOS and eNOS.^64^

Regarding the effect of metformin on EF in humans, only a few studies are available (Table 2). Rey‐Valzacchi et al.^65^ conducted a three‐arm, randomized, double‐blind, placebo‐controlled study on 64 patients with ED and insulin resistance, with poor response to sildenafil. The authors evaluated EF through IIEF‐5 and insulin resistance through homeostasis model assessment. A total of 17 patients were treated with metformin, 30 patients were treated with sulfonylureas, and the remaining 13 received placebo. After 2 months, only the first group showed an improvement of ED and insulin resistance.^65^ The authors concluded that the combination of metformin and Phosphodiesterase type 5 inhibitors (PDE5i) improves ED in patients with severe obesity and insulin resistance,^65^ probably due to the effect of metformin on insulin resistance.^65^ Indeed, PDE5i needs sufficient levels of NO to be effective, which are decreased in an insulin resistance milieu.^15^, ^66^

However, the positive effect of metformin on ED was not shown by all studies. Indeed, Al‐Kuraishy and Al‐Gareeb^8^ conducted a cross‐sectional study on patients with T2DM: 34 patients received metformin and 30 sulfonylureas; 27 men without DM were also enrolled as the control group. The authors observed that patients treated with metformin have lower levels of testosterone and sex hormone‐binding globulin blood concentrations compared with patients treated with sulfonylureas.^8^ Moreover, patients treated with metformin had worse EF (evaluated by IIEF‐5), compared with patients treated with sulfonylureas.^8^

### 3.3 Insulin

Insulin therapy is the most effective treatment for DM. Studies in diabetic rats showed an improvement of EF after insulin treatment due to its activity on an eNOS‐related pathway, although insulin alone was unable to completely restore EF in basal conditions and after the administration of PDE5i.^67^ However, the addition of simvastatin to insulin treatment completely restored basal and PDE5i‐stimulated erectile response due to the stronger activation of RhoA/Rho kinase pathway, which has a crucial role in corporal apoptosis.^67^

Improved glycemic control can be insufficient to restore EF in some conditions. In fact, some studies showed that subjects with T1DM still show endothelial dysfunction, after glycemic control has been achieved, possibly because of oxidative stress.^68^, ^69^ Oxidative stress and the persistent reduction of the antioxidant superoxide dismutase (SOD) and the interaction of advanced glycation end‐products and their receptors promote the formation of “metabolic memory.”^70^, ^71^, ^72^ The importance of the metabolic memory has been assessed in a study by Wang et al.^70^ demonstrating that insulin can improve ICP, endothelial cavernous smooth muscle cells, SOD activity, and the apoptotic index. Finally, this group also assessed that the addition of the antioxidant icariside II promotes insulin effects.

To date, only a few studies were conducted on the effect of insulin treatment in men with ED (Table 2). Maiorino et al.^73^ analyzed the EF in subjects with T1DM, aged 18–35 years, with a prevalence of ED of 37%, without any differences according to insulin regimens (multidrug injection [MDI] and continuous subcutaneous insulin infusion [CSII]). Wessells et al.^74^ observed that the prevalence of ED in T1DM was lower in patients with intensive treatment versus patients in conventional therapy; intensive insulin treatment was associated with a decreased incidence of ED in patients with microalbuminuria or non‐proliferative retinopathy, whereas no difference was observed between conventional therapy versus intensive in patients in primary prevention.

In this direction, there are a few studies in subjects using CSII. In one of these studies, the effect of insulin therapy on EF was evaluated in 164 subjects with T1DM versus 60 control subjects: insulin improved ED in the diabetic group; among this group, CSII was associated with an ED prevalence rate higher than the MDI rate (39% vs. 36%); this difference was not statistically significant.^73^ Finally, Kesavadev et al.^75^ compared CSII and MDI therapies, reporting a significant reduction in ED severity and an increase in IIEF‐5 scores in the CSII group.^75^, ^76^ In conclusion, promising results, depending also on the type of insulin administration, could be in favor of protection of EF.

### 3.4 Acarbose

Acarbose is an oral antidiabetic drug that inhibits the enterocyte α‐glucosidase enzyme, slowing glucose intestinal absorption. However, its efficacy on ED has been poorly studied. Only one study was conducted on animals and none in humans. Specifically, Oyeleye et al.^77^ observed an improvement in biomarkers of ED in diabetic rats treated with acarbose, particularly when it is associated with Moringa oleifera leaves.

### 3.5 Sulfonylureas and glinides

Sulfonylureas and glinides are oral antihyperglycemic drugs that stimulate the release of insulin from pancreatic cells, acting on adenosine triphosphate (ATP)‐sensitive potassium (K) channels.^78^

A study conducted by Ruiz Rubio et al.^79^ on horses suggested a possible role of sulfonylureas in the relaxations of corpus cavernosum, due to an effect on K‐ATP channels and penile resistance arteries. Similar results were obtained by Venkateswarlu et al.,^80^ who analyzed 215 isolated human corporeal tissue strips from 57 patients with ED, and observed an important role in the relaxation of corpus cavernosum.

Finally, in the study conducted by Al‐Kuraishy and Al‐Gareeb,^8^ previously described, patients treated with sulfonylureas obtained an elevation in testosterone levels and sexual desire as well as an amelioration of EF (Table 2).

### 3.6 Thiazolidinediones

TZDs are synthetic agonists of peroxisome proliferator‐activated receptor‐γ and are used in the treatment of T2DM, belonging to the class of insulin sensitizers. Among TZDs, the most commonly used is pioglitazone, as rosiglitazone was withdrawn in some countries because of the higher risk of myocardial infarction found in treated patients.^81^

Several studies evaluated the sexual function in diabetic and aged rats, highlighting an improvement in EF after the treatment with TZDs.^82^, ^83^, ^84^, ^85^, ^86^ In particular, Kovanecz et al.^82^, ^83^ demonstrated that the treatment with pioglitazone ameliorates the corporal veno‐occlusive dysfunction, and corporal smooth muscle detriment in type 2 diabetic rats. The effect was observed with low‐dose and long‐term treatment, with inhibition of the Rho‐kinase pathway.^83^ Furthermore, Katz et al.^84^ demonstrated a protective effect of TZDs on pelvic ganglion neurons in rats undergoing bilateral cavernosal nerve crush injury (BCNI). Similar results were obtained by Aliperti et al.^85^: specifically, they found an improvement in EF in rats, mediated by an increase in the expression of eNOS and nNOS. Finally, Heidenberg et al.^86^ also found an improvement in EF in rats with BCNI, mediated by the insulin‐like growth factor‐1 pathway.

Only one study was conducted to evaluate the effect of pioglitazone on sexual function in men^87^ (Table 2). Specifically, the authors conducted a prospective, randomized, placebo‐controlled, double‐blind trial, enrolling 38 men with ED, assessed through the IIEF‐6 as poor responders to sildenafil. Patients received pioglitazone 30 mg once daily or placebo for 9 weeks, in association with sildenafil on demand. The authors found an improvement in sexual function in patients treated with pioglitazone compared with the control group, evidenced by an increase in the IIEF‐6 total score only in the former group, but not in the latter. However, these results were not related to changes in blood glucose and testosterone levels, except for a statistically significant reduction in total cholesterol levels in the group of subjects treated with pioglitazone compared with placebo.

In conclusion, several studies are needed to evaluate the effect of pioglitazone in humans; however, the vasculoprotective and antifibrotic properties in animals are promising. In this regard, Aliperti and Hellstrom^88^ speculated that pioglitazone could be a safe and effective treatment for ED in men undergoing radical prostatectomy.

### 3.7 Dipeptidyl‐peptidase‐4 inhibitors

DPP‐4 inhibitors represent a class of oral antihyperglycemic agents widely used in the treatment of T2DM. These drugs improve glycemic control by inhibiting the activation of proteins including incretins, such as GLP‐1, which regulate meal‐induced insulin release.^89^ Furthermore, DPP‐4 inhibitors lead to an increase in SDF‐1α (stromal cell‐derived factor 1) and substance P levels that stimulate the release of endothelial progenitor cells from the bone marrow and the increase in vascular growth factor (VEGF) levels. Another substrate for DPP‐4 inhibitors is pituitary adenylate cyclase‐activating polypeptide (PACAP), a hypothalamic peptide that determines an increase in VEGF and improves endothelial function. Furthermore, PACAP promotes release of gonadotrophins and increases sex steroid levels, which play a key role in endothelial homeostasis.^90^ Therefore, DPP‐4 inhibitors carry out a protective action on the endothelium, reducing the tone of vascular muscle cells by releasing vasoactive substances such as NO. As the NO pathway is crucial for EF, a condition of endothelial dysfunction can result in ED.^91^ Despite several pieces of evidence reporting the endothelial protective role of DPP‐4 inhibitors, no studies are available regarding the effect of these drugs on EF. Altabas and Altabas^90^ hypothesized that DPP‐4 inhibitors could improve EF because of their endothelial reparatory effects. Furthermore, DPP‐4 is widely expressed in the prostate and its genetic variants would seem to be associated with the gland volume.^92^ Benign prostate hyperplasia may adversely affect sexual function due to the presence of lower urinary tract symptoms^93^ and Ilias^92^ hypothesized a possible positive outcome of DPP‐4 inhibitors on sexual function through their effect on prostate size.

In diabetic patients treated with DPP‐4 inhibitors, a possible interaction with PDE5i should be considered. In this regard, Mourad et al.^94^ conducted a study on nine healthy men treated with linagliptin 5 mg and tadalafil 20 mg. The results showed that tadalafil increased the maximum plasma concentration (Cmax) and the area under the concentration–time curve, with a consequent increase in muscle pain and QTc prolongation.

In conclusion, despite the known protective effect of DPP‐4 inhibitors on the endothelium, several studies are needed to evaluate their effect on male sexual function and their possible interactions with PDE5i.

### 3.8 Glucagon‐like peptide‐1 receptor agonists

Incretins are hormones secreted by intestinal endocrine cells in response to food intake. These are represented by GLP‐1 and glucose‐dependent insulinotropic polypeptide (GIP). GLP‐1 is secreted by L cells from the ileum and colon in response to ingestion of a mixed meal or glucose. GIP is secreted by the K cells of the duodenum in response to ingestion of glucose and lipids.^95^ GLP‐1 RAs have a large spectrum of beneficial effects. These drugs improve glycemic control through the regulation of glucose‐dependent insulin secretion protecting pancreatic beta cells without inducing hypoglycemia. Furthermore, incretins lead to body weight loss, slowing gastric emptying and inducing satiety.^96^ In addition, several cardiovascular outcomes trials have shown prominent cardiovascular protective effects of GLP‐1 RAs. Indeed, GLP‐1 RAs improve the main cardiovascular risk factors, such as dyslipidemia and arterial hypertension. GLP‐1 RAs can reduce blood pressure through direct control of endothelial function by increasing NO production.^97^ Since cardiovascular diseases share risk factors with ED, some studies have investigated the possible impact of GLP‐1 RAs on EF.^98^

Animal studies highlighted that GLP‐1 RAs have a protective effect on corpus cavernosum endothelial cells and improve the EF in diabetic rats by regulating the Akt/eNOS signaling pathway.^99^ Yuan et al.^100^ showed that liraglutide improves EF in rats with T1DM through the inhibition of the Ras homolog gene family (RhoA)–Rho‐associated protein kinase pathway, which is responsible for penile flaccidity status. These data have also been confirmed for exenatide, which appears to have a positive impact on sexual function by inhibiting the NADPH oxidase.^101^ Furthermore, liraglutide and exenatide have been correlated with the levels of Sirtuin1, which is implicated in endothelial dysfunction and in the development of diabetic micro‐ and macrovascular complications and ED.^102^

Few studies have been conducted to evaluate the effect of GLP‐1 RAs on sexual function in men (Table 2). Giagulli et al.^103^ conducted a retrospective observational study on 43 obese men with recent onset of ED and suffering from DM and hypogonadism. Patients were treated with testosterone undecanoate i.m. 1000 mg every 12 weeks plus metformin (2–3 g/day) for 1 year. In patients where the glycemic target was not reached (HbA1c > 7.5%), liraglutide 1.2 μg/day was added for an additional 12 months. All patients completed the IIEF‐15 questionnaire at baseline, after 12 months of therapy with testosterone and metformin, and after 24 months, following the addition of liraglutide. Patients treated with liraglutide reached glycemic target, experienced a weight loss as well as a significant improvement in the IIEF‐5 score compared with the group receiving only testosterone and metformin.

Furthermore, Bajaj et al.^104^ conducted an exploratory analysis to evaluate the prevalence and progression of ED in patients treated with dulaglutide once weekly in 3725 participants in the Researching Cardiovascular Events with a Weekly Incretin in Diabetes. This is a randomized, double‐blind, placebo‐controlled trial investigating cardiovascular outcomes in subjects with T2DM and a previous cardiovascular event or cardiovascular risk factors treated with dulaglutide (for a median follow‐up of 5.4 years).^105^ In a sub‐analysis, the authors evaluated the occurrence of moderate or severe ED, assessed by the IIEF‐15 questionnaire, administered at baseline, after 2 years, after 5 years, and at study end from randomization. The authors highlighted that the incidence of ED was 21.3 per 100 person‐years in the dulaglutide group and 22.0 per 100 person‐years in the placebo group (HR [hazard ratio] 0.92, *p* = 0.21). These results could be a consequence of the higher prevalence of subjects with cardiovascular disease in the first group. However, dulaglutide improved EF in a restricted group of patients with cardiovascular disease; this result suggested that ED ameliorates vascular causes that dulaglutide could restore through the improvement of endothelial cell function in the corpus cavernosum, the reduction of smooth muscle dysfunction, oxidative stress, and proinflammatory cytokine concentrations in the testes. Finally, Bajaj et al.^104^ highlighted that ED is a complication of DM and should be considered during the choice of diabetes treatment.^106^

### 3.9 Sodium‐glucose cotransporter‐2 inhibitors

SGLT‐2 inhibitors have hypoglycemic activity working through the inhibition of glucose reabsorption in the proximal convoluted tubule of the kidney, increasing glycosuria.^107^, ^108^

To date, the role of SGLT‐2 inhibitors on ED is not sufficiently investigated; in fact, only data from a study on a T2DM rat model are available, the Goto–Kakizaki rat treated with empagliflozin, with or without acute intravenous injection of sildenafil^109^: rats treated with empagliflozin showed an improvement of EF versus the placebo group, due to a better response of corpora cavernosa to the NO effect, limiting oxidative activity.

The well‐known beneficial role of SGLT‐2 on cardiovascular protection^18^ led to speculation on this effect on ED too; however, a potential detrimental effect due to a higher risk for genitourinary effect and male accessory gland inflammation should also be considered.^110^

Finally, the use of SGLT‐2 has been recognized as cause of secondary erythrocytosis of increasing relevance in general practice,^111^ mainly in association with testosterone replacement therapy.^112^

## 4 CONCLUSIONS

Erectile dysfunction is recognized as one of the complications arising from steady hyperglycemia in diabetes mellitus.^7^ The treatment of diabetes mellitus, using healthy nutrition or lifestyle and specific antihyperglycemic drugs, is the best hurdle to progression of diabetic erectile dysfunction, as for any other diabetic complications. Nevertheless, a wide knowledge gap is present with regard to whether pharmacological treatments have a direct impact on erectile function, besides ameliorating the glycemic control. In fact, this is a crucial point to reach: several studies have demonstrated the efficacy of a well‐balanced glycemic control as a key to slow down complications.

Starting with dietary habits, we reported the potentially protective or deleterious impact on erectile function, depending on the specific diet. In particular, the Mediterranean diet was effective in most studies in preserving erectile function, thanks to the extensive use of plant foods as vegetables; conversely, western diets, those high in fats and/or hypercaloric, may hamper erectile function.

With regard to antihyperglycemic drugs, a limited number of studies focused on men, and metformin was the most studied. In synthesis, a several studies showed that metformin can improve erectile function, acting on endothelial function, blood pressure, and atherosclerosis. GLP‐1 RAs were explored in some studies on subjects with erectile dysfunction, with some showing promising effects.

The main limitation of this work is the shortage of trials on humans; in this regard, the section about the use of antihyperglycemic drugs in subjects with diabetes and erectile dysfunction is short and the poor information led to speculate the useful effects of these drugs on erectile function. However, the strength of this study is represented by the fact that these speculations could highlight a gap of knowledge to fill, leading to new studies, and the role of new antidiabetic drug association could be a point to focus for future evaluations, to support a “tailor made” therapy for subjects with diabetes mellitus.

In conclusion, although there are some encouraging results in favor of their protective role on erectile function, more studies are needed for acarbose, pioglitazone, DPP‐4i, and SGLT‐2i to be recommended in diabetes mellitus patients affected with erectile dysfunction.

## FUNDING INFORMATION

This research did not receive any specific grant from funding agencies in the public, commercial, or not‐for‐profit sectors.

## CONFLICT OF INTEREST

None.

## AUTHOR CONTRIBUTIONS

*Conception and design of the study*: Giuseppe Defeudis and Rossella Mazzilli. *Acquisition of data*: Giuseppe Defeudis, Rossella Mazzilli, Alfonso Maria Di Tommaso, Virginia Zamponi, Mikiko Watanabe, and Dario Tuccinardi. *Drafting the article*: Giuseppe Defeudis, Rossella Mazzilli, Alfonso Maria Di Tommaso, Virginia Zamponi, Mikiko Watanabe, and Dario Tuccinardi. *Critical revision of the article for important intellectual content*: Giuseppe Defeudis, Rossella Mazzilli, Francesco Carlomagno, Antongiulio Faggiano, and Daniele Gianfrilli. All authors gave final approval of the version to be submitted.
